# Immunological and Cardiometabolic Risk Factors in the Prediction of Type 2 Diabetes and Coronary Events: MONICA/KORA Augsburg Case-Cohort Study

**DOI:** 10.1371/journal.pone.0019852

**Published:** 2011-06-06

**Authors:** Christian Herder, Jens Baumert, Astrid Zierer, Michael Roden, Christa Meisinger, Mahir Karakas, Lloyd Chambless, Wolfgang Rathmann, Annette Peters, Wolfgang Koenig, Barbara Thorand

**Affiliations:** 1 Institute for Clinical Diabetology, German Diabetes Center, Leibniz Center for Diabetes Research at Heinrich Heine University Düsseldorf, Düsseldorf, Germany; 2 Institute of Epidemiology II, Helmholtz Zentrum München, German Research Center for Environmental Health, Neuherberg, Germany; 3 Department of Metabolic Diseases, Heinrich Heine University Düsseldorf, Düsseldorf, Germany; 4 Department of Internal Medicine II - Cardiology, University of Ulm Medical Center, Ulm, Germany; 5 Department of Biostatistics, University of North Carolina at Chapel Hill, Chapel Hill, North Carolina, United States of America; 6 Institute of Biometrics and Epidemiology, German Diabetes Center, Leibniz Center for Diabetes Research at Heinrich Heine University Düsseldorf, Düsseldorf, Germany; University of Bremen, Germany

## Abstract

**Background:**

This study compares inflammation-related biomarkers with established cardiometabolic risk factors in the prediction of incident type 2 diabetes and incident coronary events in a prospective case-cohort study within the population-based MONICA/KORA Augsburg cohort.

**Methods and Findings:**

Analyses for type 2 diabetes are based on 436 individuals with and 1410 individuals without incident diabetes. Analyses for coronary events are based on 314 individuals with and 1659 individuals without incident coronary events. Mean follow-up times were almost 11 years. Areas under the receiver-operating characteristic curve (AUC), changes in Akaike's information criterion (ΔAIC), integrated discrimination improvement (IDI) and net reclassification index (NRI) were calculated for different models. A basic model consisting of age, sex and survey predicted type 2 diabetes with an AUC of 0.690. Addition of 13 inflammation-related biomarkers (CRP, IL-6, IL-18, MIF, MCP-1/CCL2, IL-8/CXCL8, IP-10/CXCL10, adiponectin, leptin, RANTES/CCL5, TGF-β1, sE-selectin, sICAM-1; all measured in nonfasting serum) increased the AUC to 0.801, whereas addition of cardiometabolic risk factors (BMI, systolic blood pressure, ratio total/HDL-cholesterol, smoking, alcohol, physical activity, parental diabetes) increased the AUC to 0.803 (ΔAUC [95% CI] 0.111 [0.092–0.149] and 0.113 [0.093–0.149], respectively, compared to the basic model). The combination of all inflammation-related biomarkers and cardiometabolic risk factors yielded a further increase in AUC to 0.847 (ΔAUC [95% CI] 0.044 [0.028–0.066] compared to the cardiometabolic risk model). Corresponding AUCs for incident coronary events were 0.807, 0.825 (ΔAUC [95% CI] 0.018 [0.013–0.038] compared to the basic model), 0.845 (ΔAUC [95% CI] 0.038 [0.028–0.059] compared to the basic model) and 0.851 (ΔAUC [95% CI] 0.006 [0.003–0.021] compared to the cardiometabolic risk model), respectively.

**Conclusions:**

Inclusion of multiple inflammation-related biomarkers into a basic model and into a model including cardiometabolic risk factors significantly improved the prediction of type 2 diabetes and coronary events, although the improvement was less pronounced for the latter endpoint.

## Introduction

Based on data from observational and intervention studies, subclinical inflammation is considered a risk factor for the development of type 2 diabetes [1]–[3]. In many prospective studies, circulating concentrations of acute-phase proteins, cytokines, chemokines and soluble adhesion molecules are associated with incident type 2 diabetes [1], [4]. However, the association of each of these biomarkers alone with incident disease is rather weak, because hazard ratios (HRs) for 1-SD increases of single biomarkers are usually <2 and mostly even <1.5 [5]. Statistical simulations indicate that higher HRs or the combination of multiple biomarkers with low HRs are needed for better prediction [6], [7]. Despite the wealth of data on individual inflammation-related biomarkers and incident type 2 diabetes, the predictive value of combinations of multiple of these biomarkers is still unclear [4].

Simple immune scores based on five or six markers of subclinical inflammation in the ARIC (Atherosclerosis Risk In Communities) and MONICA/KORA (MONItoring of trends and determinants in CArdiovascular disease/Cooperative Health Research in the Region of Augsburg) Augsburg cohorts showed that the risk for type 2 diabetes was almost 4-fold increased in individuals with high compared to those with low circulating levels of all tested immune markers after adjustment for multiple confounders [8], [9]. These initial data indicated that the strength of association between subclinical inflammation and incident type 2 diabetes could be increased by a combination of several inflammation-related biomarkers. However, both studies did not report areas under the receiver-operating characteristic curves (AUC) or C-statistics to assess the relevance of these biomarkers for the prediction of type 2 diabetes or to compare them with established cardiometabolic risk factors.

Two recent reports from the Inter99 and FINRISK97 cohorts provided some evidence that risk scores from multiple biomarkers may indeed improve the prediction of type 2 diabetes over and above certain established risk factors [5], [10]. Both studies included measurements of a large range of inflammation-related biomarkers. However, cardiometabolic and inflammation-related biomarkers were combined with the aim to derive final risk scores based on only a few biomarkers for diabetes prediction, whereas the question how cardiometabolic and inflammation-related biomarkers compare has not been addressed. Moreover, both studies had in common that they were based on biomarker measurements from fasting blood samples. As circulating concentrations of several immune mediators respond to food intake and/or display circadian rhythms [11], [12], it is as yet unknown whether measurements of inflammation-related biomarkers from nonfasting blood samples can also be used for modeling the risk of type 2 diabetes.

In order to characterize the relevance of markers of subclinical inflammation for the prediction of physician-diagnosed type 2 diabetes, we addressed the following questions in the population-based MONICA/KORA cohort: (i) What is the accuracy of models based on inflammation-related biomarkers in the prediction of incident type 2 diabetes? (ii) Is this accuracy comparable with established cardiometabolic risk factors? (iii) Can the accuracy be improved by combining both sets of risk factors? (iv) How does the accuracy of prediction of incident diabetes compare with that of coronary events using the same set of biomarkers and risk factors in the same population?

## Materials and Methods

### Study Design and Population

The design of this prospective case-cohort study within the population-based MONICA/KORA Augsburg cohort has been described in detail before [9], [13], [14]. Briefly, three independent cross-sectional population-based surveys were performed within the MONICA Augsburg project in 1984/85 (S1), 1989/90 (S2) and 1994/95 (S3) in Augsburg and two adjacent counties (Germany). The total number of participants was 13,427 (6,725 men, 6,702 women) aged 25–64 (S1) or 25–74 years (S2, S3). All subjects were prospectively followed within the KORA research frame. The studies were approved by the local authorities and performed according to the Declaration of Helsinki. The case-cohort study was approved by the Bayerische Landesärztekammer. All participants provided written informed consent.

The incidence of type 2 diabetes between participants' study start dates and December 31^st^, 2002 was assessed using a written follow-up questionnaire sent to all participants of the 3 baseline surveys in 1997/1998 and 2002/2003. Furthermore, all S1 participants were invited to a follow-up examination in 1987/1988. Cases with self-reported incident diabetes were validated by a questionnaire mailed to the treating physician or medical chart review [13]. Only subjects for whom the treating physician clearly reported a diagnosis of type 2 diabetes or for whom a diagnosis of type 2 diabetes was mentioned in the medical records or who were taking antidiabetic medication were classified as cases for the present analysis. Measurements of autoantibodies to exclude type 1 diabetes were not performed in the study.

Details on the selection of study participants are shown in the supporting information (Fig. S1). This study was based on 1,846 participants (244 men, 192 women with incident type 2 diabetes; 693 men, 717 women without incident type 2 diabetes) with complete information on all biomarkers and cardiometabolic risk factors and no prevalent diabetes. Mean follow-up time (± SD) was 10.6±4.6 years (range 1.0–18.2 years).

Incident coronary events were defined as combined endpoint of incident non-fatal myocardial infarction and fatal coronary death or sudden cardiac death before the age of 75 years. Cases were identified through the MONICA/KORA Augsburg coronary event registry and follow-up questionnaires for persons who had moved out of the study area. Until December 2000, a major non-fatal myocardial infarction was diagnosed based on the MONICA algorithm (symptoms, cardiac enzymes, ECG changes). Since January 2001 myocardial infarction was diagnosed according to criteria defined by the European Society of Cardiology and the American College of Cardiology [15], [16]. Coronary deaths were validated by autopsy reports, death certificates, chart review and information from the last treating physician.

Details on the selection of study participants are shown in the supporting information (Fig. S2). This study was based on 1,973 participants (239 men, 75 women with incident coronary events; 793 men, 866 women without incident coronary events) with complete information on all biomarkers and cardiometabolic risk factors and no prevalent myocardial infarction. Mean follow-up time (± SD) was 10.5±4.5 years (range 0.05–18.2 years).

### Assessment of Cardiometabolic and Inflammation-Related Risk Factors

Information on sociodemographic and lifestyle variables as well as on parental history of diabetes and myocardial infarction was collected through standardized interviews. In addition, standardized medical examinations including collection of a nonfasting venous blood sample were performed. All procedures and laboratory methods for cardiometabolic risk factors have been described in detail [9], [13], [14], [17], [18].

Laboratory methods for the assessment of inflammation-related biomarkers have been reported before [9], [13], [14], [19]–[22]. Table S1 (supporting information) provides an overview of assays, reagents and coefficients of variation (CVs) for the measurement of serum concentrations of C-reactive protein (CRP); interleukin-6 (IL-6); IL-18; macrophage-migration inhibitory factor (MIF); monocyte chemoattractant protein-1 (MCP-1)/C-C motif ligand 2 (CCL2); IL-8/C-X-C motif ligand 8 (CXCL8); interferon-γ-inducible protein-10 (IP-10)/CXCL10; adiponectin; leptin; regulated on activation, normal T-cell expressed and secreted (RANTES)/CCL5; transforming growth factor-β1 (TGF-β1); soluble E-selectin (sE-selectin); and soluble intercellular adhesion molecule-1 (sICAM-1).

### Statistical Analysis

Descriptive analyses for baseline characteristics were carried out for cases and non-cases for both outcomes. For continuous variables, means with SD were determined using the SAS procedure SURVEYMEANS and were compared with t tests using SURVEYREG. In case of non-normality, log-transformed variables were used, and results were presented as geometric means with antilogs of SEs of the adjusted log-means. For categorical variables cases and non-cases were compared using Wald chi-square test using SURVEYFREQ. Weighting was performed using the survey- and sex-specific sampling weights. Correlations between inflammation-related biomarkers were assessed by Spearman correlation coefficients (*r*).

To assess the impact of inflammation-related biomarkers and cardiometabolic risk factors on incident type 2 diabetes or coronary events, Cox proportional hazards regression was applied by calculating a first model for each biomarker which included the respective biomarker and age, sex and survey as adjustments (model 1) and a second model which included additionally the categorical variables smoking status (never/former/current smoker), alcohol consumption (no/moderate/high consumption), physical activity (low/high), family history of diabetes for incident type 2 diabetes or family history of myocardial infarction for incident coronary events (positive/negative/unknown), and prevalent diabetes for incident coronary events (yes/no), as well as the continuous variables systolic blood pressure, ratio of total cholesterol to HDL cholesterol and BMI as adjustments (model 2). Additional models were calculated by reducing or extending the number of covariables as indicated. For the Cox regression, each biomarker concentration was included standardized by subtracting the mean and divided by the SD of the biomarker concentration [(biomarker – mean(biomarker))/SD(biomarker)]. This standardization (also known as z transformation) allows a comparison of the associations of each biomarker as it is independent from the underlying unit and distribution. Based on the Cox regression, HRs with 95% confidence intervals (95% CI) and *P* values were calculated. For all analyses, *P*<0.05 was considered to be statistically significant.

The accuracy of the different models to assess 10-year event risk were estimated by four measures: (i) the area under the receiver-operating characteristic curve (AUC) (also known as C-statistic or C-index) using survival probabilities within 10 years estimated by a modified Kaplan-Meier method to account for censored observations and the weighting scheme appropriate to the case-cohort design; AUC differences between two models are given as ΔAUC [23]; (ii) Akaike's information criterion (AIC) regarding an AIC difference (ΔAIC) between two models of >10 essentially different (the lower the AIC, the better the fit of the model) [24], [25]; (iii) the integrated discrimination improvement (IDI) statistics which can be viewed as the difference of the R^2^ statistic between two models, i.e. the difference in the proportion of variance explained by the two models [26], [27]; and (iv) the net reclassification index (NRI) using the categories 0–3.0%, 3.1–8.0%, 8.1–15.0% and >15% [26], [27]. Sensitivity analyses were performed using lower (0–2.0%, 2.1–5.0%, 5.1–10.0% and >10%) and higher (0–5.0%, 5.1–10.0%, 10.1–20.0% and >20%) thresholds for both outcomes. 95% CI for ΔAUC and IDI were calculated using a bootstrap percentile approach following Efron and Tibshirani [28]. The bootstrap sampling was conducted accounting for the case-cohort design. All statistical evaluations were performed using the SAS software package (Version 9.1, SAS-Institute, Cary, NC, USA).

## Results

### Study Populations

Table S2 gives the baseline characteristics of the overlapping study populations with/without incident type 2 diabetes as well as with/without coronary events during the follow-up period. Slightly larger populations with fewer exclusions due to incomplete biomarker data have been described before [13], [29]. Briefly, cases for both outcomes were older, more likely to be male, had a higher BMI, a less favorable metabolic profile and higher levels for most inflammation-related biomarkers. A correlation matrix for the inflammation-related biomarkers is given in the supporting information (Table S3).

### Modeling the Risk of Type 2 Diabetes

**Figure 1:**
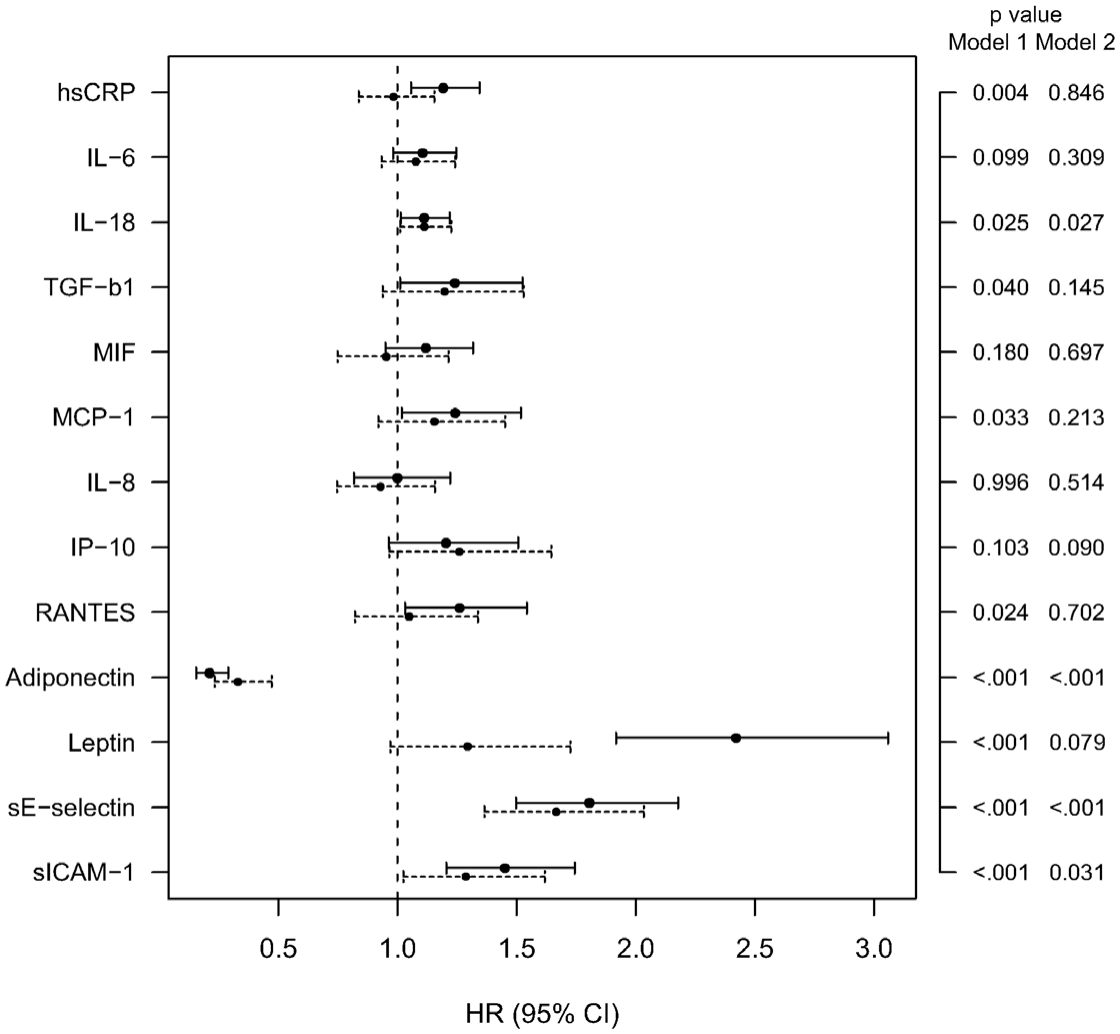
Hazard ratios (95% CI) for incident type 2 diabetes for z-transformed inflammation-related biomarkers. Solid lines (model 1): each biomarker adjusted for age, sex and survey. Dashed lines (model 2): each biomarker adjusted for age, sex, survey, BMI, systolic blood pressure, ratio of total cholesterol to HDL cholesterol, smoking, alcohol consumption, physical activity and parental diabetes.

Fig. 1 shows HRs (95% CI) for increases of inflammation-related biomarkers standardized by z-transformation for incident type 2 diabetes. In the model adjusted for age, sex, survey and cardiometabolic risk factors (model 2), IL-18, adiponectin, sE-selectin and sICAM-1 were each significantly associated with incident type 2 diabetes with HRs betwen 1.11 and 1.67 (0.33 for the protective adipokine adiponectin).

AUCs were calculated for different sets of risk factors for incident type 2 diabetes (Table 1). Age, sex and survey (model a) predicted type 2 diabetes with an AUC of 0.690 (the AUC for age alone was 0.670). Addition of all 13 inflammation-related biomarkers (model b) significantly increased the AUC to 0.801 (ΔAUC [95% CI] 0.111 [0.092–0.149] compared to model a; *P*<0.05). Similarly, addition of cardiometabolic risk factors (BMI, systolic blood pressure, ratio of total cholesterol/HDL cholesterol, smoking, alcohol, physical activity, parental history of diabetes; model c) significantly improved the AUC to 0.803 (ΔAUC [95% CI] 0.113 [0.093–0.149] compared to model a; *P*<0.05). When all 13 biomarkers were added to model c, the combination of age, sex, survey, inflammation-related biomarkers and cardiometabolic risk factors (model d) led to an AUC of 0.847 (ΔAUC [95% CI] 0.044 [0.028–0.066] compared to model c, *P*<0.05). The differences in ΔAUC, AIC as well as data for IDI and NRI (Table 1, Table S4) indicate that addition of all 13 inflammation-related biomarkers to the basic model a or to the cardiometabolic risk model c improved the models substantially.

**Table 1:** Predictive value of Cox regression models for each inflammation-related biomarker assessed by AUC for incident type 2 diabetes.

Biomarker	AUC1^a^	ΔAUC1 (95% CI)^a^	AUC2^b^	ΔAUC2 (95% CI)^b^
**None**	0.690	—	0.803	—
**hsCRP**	0.693	**0.003 (0.001–0.008)**	0.803	0.000 (−0.000–0.002)
**IL-6**	0.692	**0.002 (0.001–0.007)**	0.804	0.001 (−0.000–0.004)
**IL-18**	0.694	**0.004 (0.002–0.019)**	0.805	**0.002 (0.000–0.012)**
**TGF-β1**	0.694	**0.004 (0.001–0.011)**	0.805	0.002 (−0.000–0.007)
**MIF**	0.692	**0.002 (0.001–0.007)**	0.804	0.001 (−0.000–0.003)
**MCP-1**	0.693	**0.003 (0.001–0.010)**	0.804	0.001 (−0.000–0.006)
**IL-8**	0.691	**0.001 (0.001–0.004)**	0.803	0.000 (−0.000–0.002)
**IP-10**	0.695	**0.005 (0.001–0.016)**	0.807	0.004 (−0.000–0.010)
**RANTES**	0.695	**0.005 (0.001–0.013)**	0.803	0.000 (−0.000–0.003)
**Adiponectin**	0.753	**0.063 (0.042–0.088)**	0.826	**0.023 (0.010–0.037)**
**Leptin**	0.728	**0.038 (0.024–0.062)**	0.805	0.002 (−0.000–0.007)
**sE-selectin**	0.716	**0.026 (0.010–0.054)**	0.821	**0.018 (0.006–0.031)**
**sICAM-1**	0.699	**0.009 (0.002–0.022)**	0.806	0.003 (−0.000–0.009)
**With all 13 biomarkers**	0.801	**0.111 (0.092–0.149)**	0.847	**0.044 (0.028–0.066)**
**With IL-18, adiponectin, sE-selectin, sICAM-1** c	0.783	**0.093 (0.071–0.125)**	0.841	**0.038 (0.021–0.056)**

Bold print denotes statistical significance for ΔAUC (*P*<0.05). “−0.000” denotes values between −0.0005 and 0.0000.

a Adjusted for age, sex and survey (model 1).

b Adjusted for age, sex, survey, BMI, systolic blood pressure, ratio of total cholesterol/HDL cholesterol, smoking, alcohol, physical activity and parental history of diabetes (model 2).

c With biomarkers that were significantly associated with incident type 2 diabetes in multivariable-adjusted models (IL-18, adiponectin, sE-selectin, sICAM-1).

ΔAUC denotes the differences between the model with the respective inflammation-related biomarker and the model without any inflammation-related biomarker. ΔAUC for the difference between the model adjusted for age, sex, survey and cardiometabolic risk factors (model c) and the basic model adjusted for age, sex and survey (model a) was 0.113 [95% CI 0.093–0.149].

Measures for ΔAUC, ΔAIC, IDI and NRI also revealed that adiponectin and E-selectin were the biomarkers which improved model fit the most when added to model c. Moreover, a model that only included the four biomarkers that were significantly associated with incident diabetes (Fig. 1) was almost as good as model d based on all 13 biomarkers (Table 1, Table S4).

### Modeling the Risk of Coronary Events

**Figure 2:**
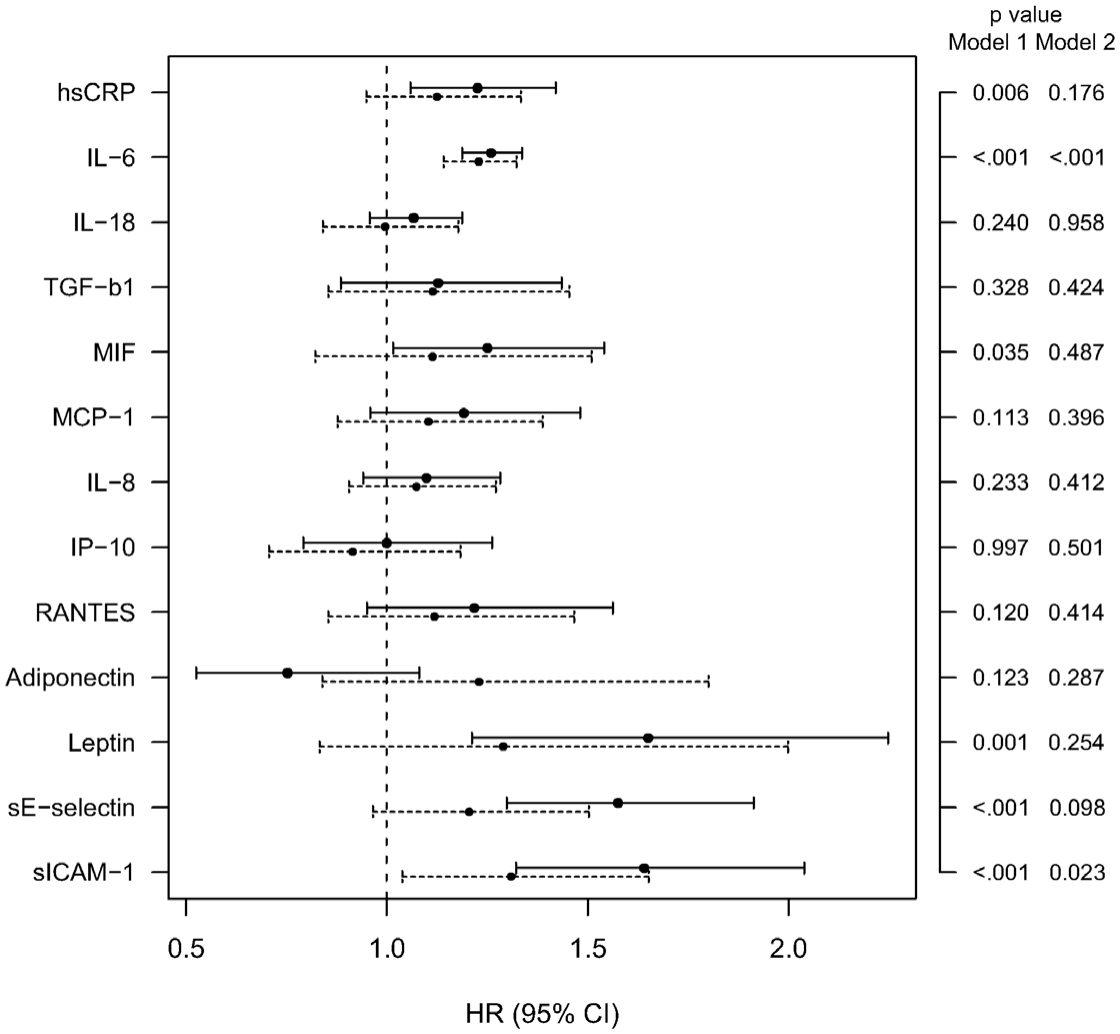
Hazard ratios (95% CI) for incident coronary events for z-transformed inflammation-related biomarkers. Solid lines (model 1): each biomarker adjusted for age, sex and survey. Dashed lines (model 2): each biomarker adjusted for age, sex, survey, BMI, systolic blood pressure, ratio of total cholesterol to HDL cholesterol, smoking, alcohol consumption, physical activity, parental myocardial infarction and prevalent diabetes.

As shown in Fig. 2, two inflammation-related biomarkers were significantly associated with the risk for coronary events in the fully adjusted models (HRs for IL-6 and sICAM-1: 1.23 and 1.31, respectively).

Analogous analyses for coronary events as described above yielded AUCs of 0.745 for age alone, 0.807 for model a (age, sex, survey), 0.825 for model b (addition of inflammation-related biomarkers to model a, ΔAUC [95% CI] 0.018 [0.013–0.038], *P*<0.05), 0.845 for model c (addition of cardiometabolic risk factors to model a, ΔAUC [95% CI] 0.038 [0.028–0.059], *P*<0.05) and 0.851 for model d (combination of all biomarkers and risk factors, ΔAUC [95% CI] 0.006 [0.003–0.021], *P*<0.05 compared with model c). The differences in ΔAUC, IDI and NRI (Table 2, Table S5) indicated that addition of all 13 inflammation-related biomarkers significantly improved the basic model a and the cardiometabolic risk model c, but improvements were much lower than for type 2 diabetes.

**Table 2:** Predictive value of Cox regression models for each inflammation-related biomarker assessed by AUC for incident coronary events.

Biomarker	AUC1^a^	ΔAUC1 (95% CI)^a^	AUC2^b^	ΔAUC2 (95% CI)^b^
**None**	0.807	—	0.845	—
**hsCRP**	0.809	**0.002 (0.001–0.008)**	0.845	0.000 (−0.001–0.003)
**IL-6**	0.810	**0.003 (0.001–0.008)**	0.846	0.001 (−0.000–0.004)
**IL-18**	0.808	**0.001 (0.001–0.005)**	0.845	0.000 (−0.001–0.003)
**TGF-β1**	0.808	**0.001 (0.001–0.005)**	0.845	0.000 (−0.000–0.003)
**MIF**	0.809	**0.002 (0.001–0.008)**	0.845	0.000 (−0.000–0.003)
**MCP-1**	0.808	**0.001 (0.001–0.005)**	0.845	0.000 (−0.001–0.002)
**IL-8**	0.808	**0.001 (0.001–0.003)**	0.845	0.000 (−0.000–0.002)
**IP-10**	0.808	**0.001 (0.000–0.005)**	0.845	0.000 (−0.001–0.005)
**RANTES**	0.808	**0.001 (0.001–0.006)**	0.845	0.000 (−0.000–0.003)
**Adiponectin**	0.808	**0.001 (0.000–0.007)**	0.845	0.000 (−0.000–0.005)
**Leptin**	0.811	**0.004 (0.001–0.010)**	0.845	0.000 (−0.000–0.004)
**sE-selectin**	0.817	**0.010 (0.004–0.018)**	0.847	0.002 (−0.000–0.009)
**sICAM-1**	0.817	**0.010 (0.005–0.021)**	0.848	**0.003 (0.000–0.011)**
**With all 13 biomarkers**	0.825	**0.018 (0.013–0.038)**	0.851	**0.006 (0.003–0.021)**
**With IL-6,sICAM-1** c	0.819	**0.012 (0.006–0.024)**	0.849	**0.004 (0.001–0.012)**

Bold print denotes statistical significance for ΔAUC (*P*<0.05). “−0.000” denotes values between −0.0005 and 0.0000.

a Adjusted for age, sex and survey (model 1).

b Adjusted for age, sex, survey, BMI, systolic blood pressure, ratio of total cholesterol/HDL cholesterol, smoking, alcohol, physical activity, parental myocardial infarction and prevalent diabetes (model 2).

c With biomarkers that were significantly associated with incident coronary events in multivariable-adjusted models (IL-6, sICAM-1).

ΔAUC denotes the differences between the model with the respective inflammation-related biomarker and the model without any inflammation-related biomarker. ΔAUC for the difference between the model adjusted for age, sex, survey and cardiometabolic risk factors (model c) and the basic model adjusted for age, sex and survey (model a) was 0.038 [95% CI 0.026–0.055].

### Sensitivity Analyses

Most inflammation-related biomarkers were measured using high-sensitivity ELISA kits with inter- and intraassay CVs generally below 10%, whereas inter- and intraassay CVs for some parameters measured with bead-based multiplex assays exceeded 10% (Table S1). In order to exclude effects of less robust assays on the estimation of model accuracy, we repeated our analyses after exclusion of the four biomarkers that were measured with the multiplex assay (i.e. IL-18, MCP-1/CCL2, IL-8/CXCL8 and IP-10/CXCL10). For incident type 2 diabetes, the AUCs for models b and d were only slightly altered (from 0.801 to 0.796 and from 0.847 to 0.843, respectively). Similar results were seen for incident coronary events, where the AUCs for models b and d changed from 0.825 to 0.826 and from 0.851 to 0.852, respectively.

We also performed sensitivity analyses replacing BMI with waist circumference in our risk models. Unfortunately, waist circumference was only measured in S2 und S3, but not in S1. Thus, our study samples were reduced to 311 cases and 928 non-cases for the outcome incident type 2 diabetes and to 219 cases and 1,133 non-cases for the outcome incident coronary events. For type 2 diabetes, the AUCs for models c and d were increased by 0.010 and 0.007, respectively. For coronary events, the increases were even lower with 0.002 for both models c and d. These results indicate that the substitution of BMI with waist circumference in this subpopulation of the case-cohort study only slightly improved the model accuracy.

Low socioeconomic status represents an important risk factor for type 2 diabetes and coronary events. Education (in years) is the only index of socioeconomic status that is available for all participants of the MONICA/KORA case-cohort study. When education was added as a covariable to the respective models c (cardiometabolic risk models), this variable was not significantly associated with incident type 2 diabetes (*P* = 0.184) or incident coronary events (*P* = 0.859) so that we decided not to include education in the list of cardiometabolic risk factors.

It has been reported that measures of NRI depend on the chosen cut-off values [30]. Our initial cut-off values (3%, 8%, 15%) were based on previous publications [26], [27], but at least for incident type 2 diabetes, a clear clinical basis for these cut-off values does not exist. Therefore, we repeated our analyses with lower (2%, 5%, 10%) and higher (5%, 10%, 20%) cut-off values. As shown in Table S6, we found minor changes for NRI values when lower or higher cut-offs were used, but overall, our results seem relatively robust. Irrespective of the used cut-off values, NRI values were always considerably higher for type 2 diabetes than for coronary events.

## Discussion

The main findings of our study are (i) that the combination of 13 biomarkers of subclinical inflammation improved the accuracy of a risk model of incident type 2 diabetes in the MONICA/KORA cohort significantly and equally well as a combination of established cardiometabolic risk factors, (ii) that a combination of both sets of risk factors led to a further significant improvement of the accuracy of predicting type 2 diabetes compared with either set of risk factors alone, and (iii) that the improvement of accuracy of prediction models for type 2 diabetes over and above age, sex and cardiometabolic risk factors by the combination of inflammation-related biomarkers was more pronounced than for coronary events employing the same methods in the same population.

The study extends previous knowledge because it is the first study to focus on the predictive value of multiple markers of subclinical inflammation for incident type 2 diabetes. The set of 13 inflammation-related biomarkers consists of an acute-phase protein (CRP), cytokines (IL-6, IL-18, TGF-β1, MIF), chemokines (MCP-1/CCL2, IL-8/CXCL8, IP-10/CXCL10, RANTES/CCL5), adipokines (adiponectin, leptin) and soluble adhesion molecules (sE-selectin, sICAM-1) and is therefore more comprehensive than the combinations of immune mediators that were used in the ARIC cohort (leukocyte count, IL-6, four acute-phase proteins) or the MONICA/KORA cohort (CRP, IL-6, three chemokines) before [8], [9].

Our study design differs in two important aspects from the design of the aforementioned Inter99 and FINRISK cohorts [5], [10]. First, we provide an estimate of the accuracy of models based on biomarkers of subclinical inflammation only (over and above age, sex and survey as essential covariates) and both compared and combined them with established cardiometabolic risk factors because we were interested in the contribution of subclinical inflammation as pathophysiological mechanism to the development of type 2 diabetes. Therefore, it was not our aim to build a risk score with optimal predictive value for incident type 2 diabetes. Second, we used nonfasting rather than fasting blood samples as fasting samples were not available from the MONICA/KORA Augsburg surveys. Although it could be argued that this represents a major limitation of our study (to be discussed below), it should be noted that the question whether inflammation-related biomarkers from nonfasting samples could be useful in the prediction of type 2 diabetes has not been addressed in comparable population-based studies and is therefore of interest.

Although we observed a substantial increase in AUC (0.044 [95% CI 0.028–0.066]) as well as large values for ΔAIC (139.8), IDI (0.061) and NRI (0.202) by the addition of inflammation-related biomarkers to a model that already contained strong cardiometabolic risk factors for type 2 diabetes, we found that this increase could be attributed to just a few biomarkers. Only adiponectin and sE-selectin increased the AUC by more than 0.010 and showed ΔAIC considerably larger than 10. Importantly, adiponectin was also one out of four biomarkers (next to apolipoprotein B, CRP and ferritin) that was included in the final prediction score derived from 31 biomarkers in the FINRISK97 cohort [5]. Moreover, inclusion of adiponectin in an extensive risk score based on anthropometric, metabolic and lifestyle factors led to a small, but significant increase in the AUC in the EPIC-Potsdam Study [31]. We reported before that adiponectin improved risk prediction over and above cardiometabolic and selected inflammation-derived biomarkers in the MONICA/KORA Augsburg case-cohort study [22]. These data are in contrast with findings from the KORA S4/F4 cohort study, which was conducted later and independently from the MONICA/KORA surveys 1–3. In KORA S4/F4, there was no significant improvement of the AUC when adiponectin was added to a model that contained HbA1c and fasting glucose [32]. Data on the impact of sE-selectin on measures of discrimination are available from a small nested case-control study within the Western New York Study. The addition of sE-selectin, serum albumin and leukocyte count improved the accuracy of a risk model for type 2 diabetes compared to a basic model based on sex, BMI and familiy history of type 2 diabetes [33].

Our findings on type 2 diabetes are in contrast to several other studies on the improvement by inflammation-related biomarkers of risk models already containing measure of glycemia or insulin resistance. In the Insulin Resistance Atherosclerosis Study, the addition of CRP to a prediction model for type 2 diabetes that was based on the metabolic syndrome (without or with an estimate of insulin resistance) had little impact on AUCs [34]. CRP (alone or in combination with other biomarkers) also failed to improve AUCs of prediction models already containing plasma glucose glucose levels as in the Framingham Offspring Study [35] and the aforementioned EPIC-Potsdam Study [31]. In the Sandy Lake Health and Diabetes Project, leptin, CRP, IL-6 and serum amyloid A were included in a risk model based on cardiometabolic risk factors, adiponectin and impaired glucose tolerance, but could not improve diabetes prediction [36]. Recently, the Women's Health Initiative Observational Study did not find that biomarkers of subclinical inflammation (hsCRP, IL-6, soluble tumor necrosis factor-receptor 2) and of endothelial dysfunction (E-selection, ICAM-1, vascular cell adhesion molecule-1) contribute to the prediction of incident type 2 diabetes over and above clinical risk factors and fasting glucose [37]. Taken together, these data suggest that our findings may be specific for analyses based on nonfasting blood samples and that the contribution of multiple inflammation-related biomarkers to prediction models with diabetes risk factors that are used for the diagnosis of type 2 diabetes (glucose, HbA1c) may be less pronounced than for prediction models without these measures of glycemia.

An important aspect of our study is the fact that our case-cohort design allowed us to compare inflammation-related and cardiometabolic risk factors for both type 2 diabetes and coronary events as outcomes using the same methods and two largely overlapping study populations. The combination of all biomarkers and risk factors yielded almost identical AUCs for both outcomes. However, the improvement of inflammation-related biomarkers over a basic model based on age, sex and survey was considerably larger for type 2 diabetes (ΔAUC 0.111 [95% 0.092–0.149]) than for coronary events (ΔAUC 0.018 [95% CI 0.013–0.038]). This difference is confirmed by larger ΔAIC, IDI and NRI values when models for both outcomes were compared. This is most likely attributable to the higher accuracy of the basic model for coronary events (AUC 0.807) compared to type 2 diabetes (AUC 0.690) so that further improvements by additional biomarkers or risk factors can be expected to be less pronounced. Although we found a significant increase in AUC, these data are in line with data from other studies that focused on risk models for incident coronary events or cardiovascular death and that assessed the incremental predictive value of inflammation-related biomarkers. AUCs for prediction models based on cardiometabolic factors were usually in the range between 0.70 and 0.82. Although multiple promising biomarker candidates were tested, the improvement of risk models by addition of novel inflammation-related biomarkers was relatively small, especially when the basic model already had a good accuracy, and AUCs of the extended models did not increase beyond 0.82 in these studies [38]–[45]. A recent study indicated that in particular N-terminal pro-brain natriuretic peptide (NT-proBNP) and sensitive troponin I may improve the prediction of risk of coronary events [44].

Regarding the clinical relevance of our findings, the present study did not aim at providing a simple clinical risk score, but rather at studying to which extent subclinical inflammation as one of several other mechanisms contributes to the prediction of the development of type 2 diabetes. The approach of this study was chosen to extend previous work that mainly evaluated statistical associations between inflammation-related biomarkers and incident diabetes using Cox regression models.

Overall, our data demonstrate that age (but not sex or survey) contribute a substantial part to the AUC that can be achieved with a basic risk model and with more sophisticated models involving multiple risk factors and biomarkers. Interestingly, although cardiometabolic risk factors are strongly associated with inflammation-related biomarkers, we found a significant increase in accuracy when adding inflammation-related biomarkers to a model based on age, sex and cardiometabolic risk factors. Therefore, these data are in line with a role for subclinical inflammation in the development of type 2 diabetes and indicate that in particular adiponectin and sE-selectin should be further evaluated as markers for type 2 diabetes risk in combination with other risk factors and biomarkers.

Strengths of our study include the use of the MONICA/KORA Augsburg cohort with a large number of cases and non-cases, a long follow-up period, availability of data for multiple biomarkers representing different aspects of subclinical inflammation, and the inclusion of both cases with incident type 2 diabetes and coronary events in the case-cohort study so that a direct comparison of risk factors and biomarkers for both outcomes in the same cohort using the same methods was possible.

There are also several limitations that should be pointed out. First, we did not perform oral glucose tolerance tests at baseline or follow-up so that some misclassification may have occurred and the outcome of our study was physician-diagnosed type 2 diabetes. Second, we had no data on fasting glucose and fasting insulin (HbA1c data for a subgroup of study participants only) so that we could not investigate the change in AUC by inflammation-related biomarkers over a model that contained these variables. In addition, minor variations of levels of inflammation-related biomarkers due to the nonfasting state cannot be excluded. Third, we used continuous values of biomarker concentrations in order to render results comparable to other studes [5], [44], although consideration of sex differences or non-linear associations between biomarkers and endpoints could have led to higher accuracy of our models. Fourth, biomarkers of non-alcoholic fatty liver disease (liver enzymes such as alanine aminotransferase, aspartate aminotransferase or γ-glutamyl transferase) are relevant risk factors for type 2 diabetes [4], [46], but were not available in our study (with the exception of γ-glutamyl transferase in S1) so that we could not include them in our cardiometabolic risk models. Finally, we did not seek for external replication of our results.

Taken together, 13 inflammation-related biomarkers measured in nonfasting serum samples significantly improved the prediction of incident type 2 diabetes and coronary events over and above cardiometabolic risk factors in the MONICA/KORA study, but this improvement was much more pronounced for type 2 diabetes. Our study could not address the question whether biomarkers of subclinical inflammation can also improve the predictive value of risk models that contain various measures of glycemia. Therefore, further research is warranted to investigate whether multiple inflammation-related biomarkers can increase the accuracy of risk models that include data on (fasting or nonfasting) glucose, insulin or HbA1c levels.

## Supporting Information

Figure S1 Selection of incident type 2 diabetes cases and non-cases from the participants of the MONICA/KORA surveys 1, 2 and 3.
(TIF)

Figure S2 Selection of incident coronary events (CHD cases) and non-cases from the participants of the MONICA/KORA surveys 1, 2 and 3.
(TIF)

Table S1 Assays, reagents and coefficients of variation for the measurement of serum concentrations on inflammation-related biomarkers.
(DOC)

Table S2 Baseline characteristics for study participants with and without incident type 2 diabetes and with and without incident coronary events.
(DOC)

Table S3 Correlation of inflammation-related biomarkers (Spearman correlation coefficients *r*) in the randomly sampled subcohort (n = 1,795).
(DOC)

Table S4 Predictive value of Cox regression models for each inflammation-related biomarker assessed by ΔAIC, IDI and NRI for incident type 2 diabetes.
(DOC)

Table S5 Predictive value of Cox regression models for each inflammation-related biomarker assessed by ΔAIC, IDI and NRI for incident coronary events.
(DOC)

Table S6 NRI for inflammation-related biomarkers added to prediction models for incident type 2 diabetes and incident coronary events.
(DOC)
