# Supplementary figures and images for: Immunological and Cardiometabolic Risk Factors in the Prediction of Type 2 Diabetes and Coronary Events: MONICA/KORA Augsburg Case-Cohort Study
Source: PLoS One. 2011 Jun 6;6(6):e19852. doi: 10.1371/journal.pone.0019852 (PMC3108947; doi:10.1371/journal.pone.0019852)

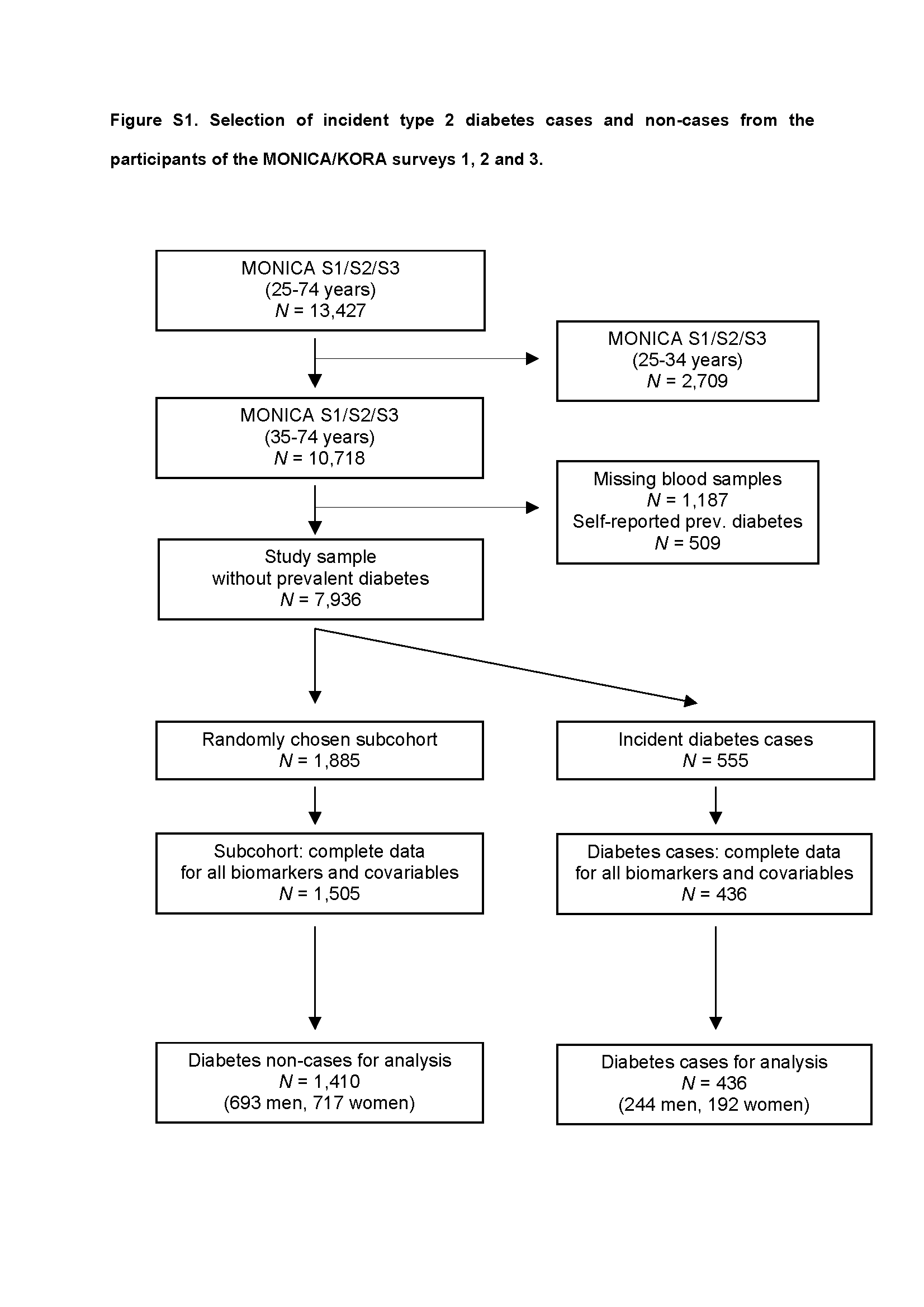

Supplement: Figure S1 — Selection of incident type 2 diabetes cases and non-cases from the participants of the MONICA/KORA surveys 1, 2 and 3. (TIF) [file pone.0019852.s001.tif]

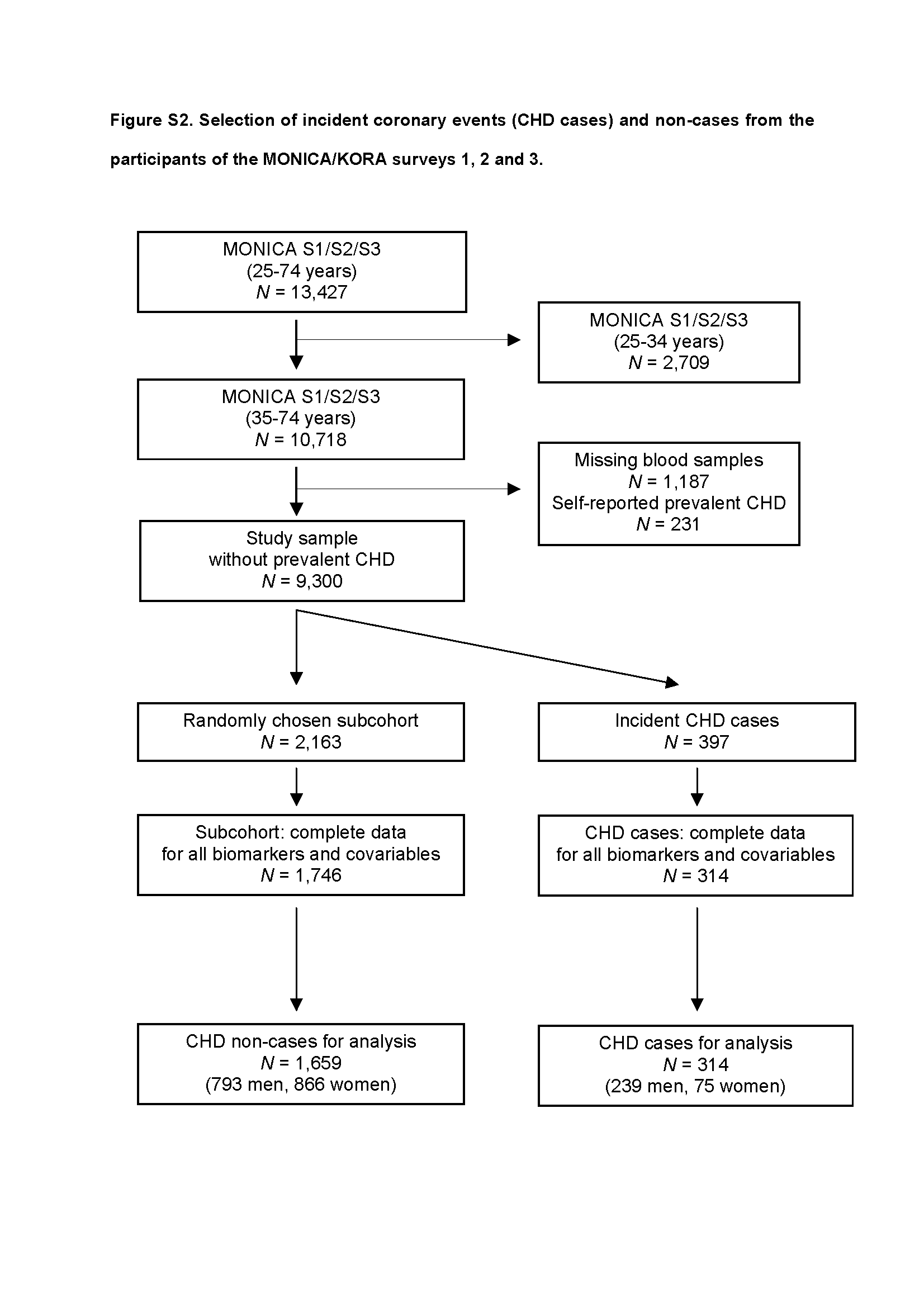

Supplement: Figure S2 — Selection of incident coronary events (CHD cases) and non-cases from the participants of the MONICA/KORA surveys 1, 2 and 3. (TIF) [file pone.0019852.s002.tif]
