# Supplementary material for: Immunological and Cardiometabolic Risk Factors in the Prediction of Type 2 Diabetes and Coronary Events: MONICA/KORA Augsburg Case-Cohort Study
Source: PLoS One. 2011 Jun 6;6(6):e19852. doi: 10.1371/journal.pone.0019852 (PMC3108947; doi:10.1371/journal.pone.0019852)
Supplement: Table S1 — Assays, reagents and coefficients of variation for the measurement of serum concentrations on inflammation-related biomarkers. (DOC) [file pone.0019852.s003.doc]

**Table S1.** Assays, reagents and coefficients of variation for the measurement of serum concentrations on inflammation-related biomarkers.

| **Biomarker** | **Assay** | **Reagents** | **Intra-/ inter-assay CV (%)** | **References** |
| --- | --- | --- | --- | --- |
| **Acute-phase protein** | | | | |
| High-sensitivity CRP | IRMA, high-sensitivity latex-enhanced nephelometric assay | BN II analyser (Dade Behring, Marburg, Germany) | 4 / 12 (IRMA),  2.5 / 5.1 (neph.) | 1, 2 |
| Cytokines | | | | |
| IL-6 | ELISA | PeliKine Compact human IL-6 ELISA (CLB, Amsterdam, The Netherlands) | <10 / <10 | 2, 3 |
| IL-18 | Bead-based multiplex assay | Fluorescent xMAP COOH microspheres (Luminex Corporation, Austin, TX, USA), recombinant MCP-1 and antibodies (MBL, Nagoya, Japan) | <10 / 14.4 | 4, 5 |
| TGF-β1 | ELISA | Quantikine Human TGF-β1 Immunoassay (R&D Systems, Wiesbaden, Germany) | 3.0 / 8.8 | 6 |
| MIF | ELISA | Human MIF Quantikine ELISA (R&D Systems) | 3.8 / 11.1 | 7, 8 |
| Chemokines | | | | |
| MCP-1/CCL2 | Bead-based multiplex assay | Fluorescent xMAP COOH microspheres (Luminex Corporation), recombinant MCP-1 and antibodies (R&D Systems) | <10 / 19.9 | 1, 9 |
| IL-8/CXCL8 | Bead-based multiplex assay | Fluorescent xMAP COOH microspheres (Luminex Corporation), recombinant IL-8 (NIBSC, Potters Bar, UK), antibodies (R&D Systems) | <10 / 10.9 | 1, 9 |
| IP-10/CXCL10 | Bead-based multiplex assay | Fluorescent xMAP COOH microspheres (Luminex Corporation), recombinant IP-10 and antibodies (BD Biosciences, Heidelberg, Germany) | <10 / 35.1 | 1, 9 |
| RANTES/CCL5 | ELISA | Human CCL5/RANTES Quantikine ELISA (R&D Systems) | 4.7 / 6.4 | 10 |
| Adipokines | | | | |
| Adiponectin | ELISA | Adiponectin ELISA (Mercodia, Uppsala, Sweden) | <10 / <10 | 11 |
| Leptin | ELISA | Leptin ELISA (Mercodia) | <10 / <10 | 11 |
| Soluble adhesion molecules | | | | |
| sE-selectin | ELISA | Human sE-Selectin/CD62E Quantikine ELISA (R&D Systems) | 3.3 / 6.3 | 12 |
| sICAM-1 | ELISA | Human sICAM-1-ELISA (Diaclone, Besançon, France) | 2.3 / 4.7 | 12 |

## Abbreviations

CCL, C-C motif ligand; CXCL, C-X-C motif ligand; ELISA, enzyme-linked immunosorbent assay; hsCRP, high-sensitivity C-reactive protein; ICAM, intercellular adhesion molecule; IL, interleukin; IP, interferon-γ-inducible protein; IRMA, high-sensitivity immunoradiometric assay; MCP, monocyte chemoattractant protein; MIF, macrophage migration inhibitory factor; RANTES, regulated on activation, normal T-cell expressed and secreted; s, soluble; TGF, transforming growth factor.

## References

1. Herder C, Baumert J, Thorand B, Koenig W, de Jager W, et al. (2006) Chemokines as risk factors for type 2 diabetes: results from the MONICA/KORA Augsburg Study, 1984-2002. Diabetologia 49: 921-929.

2. Thorand B, Baumert J, Koenig W, Kolb H, Meisinger C, et al. (2007) Sex differences in the prediction of type 2 diabetes by inflammatory markers: results from the MONICA/KORA Augsburg case-cohort study, 1984-2002. Diabetes Care 30: 854-860.

3. Müller S, Martin S, Koenig W, Hanifi-Moghaddam P, Rathmann W, et al. (2002) Impaired glucose tolerance is associated with increased serum concentrations of interleukin 6 and co-regulated acute-phase proteins but not TNF-α or its receptors. Diabetologia 45: 805-812.

4. Thorand B, Kolb H, Baumert J Koenig W, Chambless L, et al. (2005) Elevated levels of interleukin-18 predict the development of type 2 diabetes: results from the MONICA/KORA Augsburg Study 1984-2002. Diabetes 54: 2932-2938.

5. Koenig K, Khuseyinova N, Baumert J, Thorand B, Loewel H, et al. (2006) Increased concentrations of C-reactive protein and IL-6 but not IL-18 are independently associated with incident coronary events in middle-aged men and women. Results from the MONICA/KORA Augsburg Case-Cohort Study, 1984-2002. Arterioscler Thromb Vasc Biol 26: 2745-2751.

6. Herder C, Zierer A, Koenig W, Roden M, Meisinger C, et al. (2009) Transforming growth factor-β1 and incident type 2 diabetes. Results from the MONICA/KORA case-cohort study, 1984-2002. Diabetes Care 32: 1921-1923.

7. Herder C, Klopp N, Baumert J, Müller M, Khuseyinova N, et al. (2008) Effect of macrophage migration inhibitory factor (MIF) gene variants and MIF serum concentrations on the risk of type 2 diabetes: results from the MONICA/KORA Augsburg Case-Cohort Study, 1984-2002. Diabetologia 51: 276-284.

8. Herder C, Illig T, Baumert J, Müller M, Klopp N, et al. (2008) Macrophage migration inhibitory factor (MIF) and risk for coronary heart disease: Results from the MONICA/KORA Augsburg case-cohort study, 1984-2002. Atherosclerosis 200: 380-388.

9. Herder C, Baumert J, Thorand B, Martin S, Löwel H, et al. (2006) Chemokines and incident coronary heart disease: results from the MONICA/KORA Augsburg Case-Cohort Study, 1984-2002. Arterioscler Thromb Vasc Biol 26: 2147-2152.

10. Herder C, Illig T, Baumert J, Müller M, Klopp N, et al. (2008) RANTES/*CCL5* gene polymorphisms, serum concentrations and incident type 2 diabetes: results from the MONICA/KORA Augsburg case-cohort study, 1984-2002. Eur J Endocrinol 158: R1-R5.

11. Thorand B, Zierer A, Baumert J, Meisinger C, Herder C, et al. (2010) Associations between leptin and the leptin/adiponectin ratio and incident type 2 diabetes in middle-aged men and women: results from the MONICA/KORA Augsburg Study 1984-2002. Diabet Med 27: 1004-1011.

12. Thorand B, Baumert J, Chambless L, Meisinger C, Kolb H, et al. (2006) Elevated markers of endothelial dysfunction predict type 2 diabetes mellitus in middle-aged men and women from the general population. Arterioscler Thromb Vasc Biol 26: 398-405.
