# Supplementary material for: Immunological and Cardiometabolic Risk Factors in the Prediction of Type 2 Diabetes and Coronary Events: MONICA/KORA Augsburg Case-Cohort Study
Source: PLoS One. 2011 Jun 6;6(6):e19852. doi: 10.1371/journal.pone.0019852 (PMC3108947; doi:10.1371/journal.pone.0019852)
Supplement: Table S2 — Baseline characteristics for study participants with and without incident type 2 diabetes and with and without incident coronary events. (DOC) [file pone.0019852.s004.doc]

**Table S2.** Baseline characteristics for study participants with and without incident type 2 diabetes and with and without incident coronary events.

|  | Incident type 2 diabetes | | | Incident coronary events | | |
| --- | --- | --- | --- | --- | --- | --- |
| Characteristics | Cases | Non-cases | *P* | Cases | Non-cases | *P* |
| Number | 436 | 1,410 | - | 314 | 1,659 | - |
| Age (years) *^a^* | 56.81 (0.47) | 51.86 (0.29) | <0.001 | 57.61 (0.46) | 52.50 (0.27) | <0.001 |
| Male sex (%) | 56.0 | 45.3 | <0.001 | 76.1 | 44.4 | <0.001 |
| Education < 12 years (%) | 83.0 | 75.2 | <0.001 | 78.3 | 77.1 | 0.639 |
| Anthropometry |  |  |  |  |  |  |
| Body mass index (kg/m^2^) ^a^ | 30.31 (0.22) | 26.74 (0.11) | <0.001 | 28.43 (0.24) | 27.06 (0.11) | <0.001 |
| Metabolic variables |  |  |  |  |  |  |
| Systolic BP (mm Hg) ^a^ | 142.92 (0.92) | 132.77 (0.54) | <0.001 | 143.05 (1.18) | 133.74 (0.49) | <0.001 |
| Diastolic BP (mm Hg) ^a^ | 84.75 (0.55) | 81.32 (0.31) | <0.001 | 83.28 (0.69) | 81.41 (0.28) | 0.012 |
| Actual hypertension (%) | 69.5 | 39.5 | <0.001 | 66.9 | 41.4 | <0.001 |
| Ratio TC/HDL-C ^a^ | 5.55 (0.11) | 4.44 (0.05) | <0.001 | 5.78 (0.12) | 4.49 (0.04) | <0.001 |
| Prevalent diabetes | - | - | - | 18.8 | 4.7 | <0.001 |
| Lifestyle factors |  |  |  |  |  |  |
| Level of low physical activity (%) | 69.5 | 58.8 | <0.001 | 72.3 | 61.3 | <0.001 |
| Smoking status (%) |  |  |  |  |  |  |
| Never smoker | 42.7 | 49.5 | 0.025 | 27.1 | 49.6 | <0.001 |
| Former smoker | 33.5 | 27.3 |  | 33.8 | 27.4 |  |
| Current smoker | 23.9 | 23.3 |  | 39.2 | 23.0 |  |

| Alcohol intake (%) |  |  |  |  |  |  |
| --- | --- | --- | --- | --- | --- | --- |
| 0 g/day | 35.8 | 29.9 | 0.073 | 29.9 | 32.7 | 0.366 |
| 0.1 – 39.9/19.9 g/day ^b^ | 39.2 | 44.0 |  | 41.1 | 42.1 |  |
| ≥ 40/20 g/day ^b^ | 25.0 | 26.1 |  | 29.0 | 25.2 |  |
| Parental history of diabetes or MI (%) |  |  |  |  |  |  |
| Negative | 45.9 | 59.3 | <0.001 | 50.6 | 58.3 | 0.043 |
| Positive | 28.0 | 20.3 |  | 23.2 | 20.5 |  |
| Unknown | 26.1 | 20.3 |  | 26.1 | 21.1 |  |
| Current HRT (%) ^c^ | 9.3 | 11.9 | 0.377 | 6.3 | 10.5 | 0.225 |
| Current use of OC (%) ^d^ | 7.1 | 15.0 | 0.090 | 0.0 | 14.2 | NA |
| Inflammation-related biomarkers |  |  |  |  |  |  |
| CRP (mg/l) ^e^ | 2.53 (1.05) | 1.36 (1.03) | <0.001 | 2.60 (1.06) | 1.44 (1.03) | <0.001 |
| IL-6 (pg/ml) ^e^ | 3.02 (1.04) | 1.97 (1.03) | <0.001 | 3.20 (1.05) | 2.00 (1.03) | <0.001 |
| IL-18 (pg/ml) ^e^ | 189.63 (1.03) | 158.23 (1.02) | <0.001 | 172.21 (1.04) | 160.13 (1.02) | 0.125 |
| TGF-β1 (ng/ml) ^e^ | 34.87 (1.01) | 34.22 (1.01) | 0.129 | 34.64 (1.01) | 34.23 (1.01) | 0.373 |
| MIF (ng/ml) ^e^ | 18.27 (1.02) | 17.93 (1.01) | 0.390 | 18.96 (1.02) | 18.15 (1.01) | 0.071 |
| MCP-1/CCL2 (pg/ml) ^e^ | 207.29 (1.04) | 177.88 (1.02) | 0.001 | 214.20 (1.05) | 178.29 (1.02) | <0.001 |
| IL-8/CXCL8 (pg/ml) ^e^ | 7.95 (1.03) | 6.97 (1.02) | <0.001 | 8.59 (1.04) | 6.97 (1.02) | <0.001 |
| IP-10/CXCL10 (pg/ml) ^e^ | 250.24 (1.04) | 213.23 (1.02) | <0.001 | 240.23 (1.04) | 217.18 (1.02) | 0.033 |
| RANTES (pg/ml) ^e^ | 24700 (1.03) | 23169 (1.02) | 0.052 | 25044 (1.03) | 23211 (1.01) | 0.029 |
| Adiponectin (µg/ml) ^e^ | 9.37 (1.02) | 11.52 (1.01) | <0.001 | 10.07 (1.02) | 11.47 (1.01) | <0.001 |
| Leptin (µg/l) ^e^ | 14.26 (1.05) | 9.11 (1.03) | <0.001 | 7.90 (1.06) | 9.73 (1.03) | 0.001 |
| sE-selectin (ng/ml) ^a^ | 71.45 (2.00) | 54.01 (0.71) | <0.001 | 67.22 (2.25) | 55.13 (0.67) | <0.001 |
| sICAM-1 (ng/ml) ^a^ | 849.06 (14.91) | 754.24 (7.85) | <0.001 | 887.58 (20.08) | 758.47 (7.22) | <0.001 |

Abbreviations: BP, blood pressure; TC, total cholesterol; HDL-C, HDL cholesterol; CRP, C-reactive protein; sE-selectin, soluble E-selectin; sICAM-1, soluble ICAM-1; HRT, hormone replacement therapy; MI, myocardial infarction; NA, not applicable; OC, oral contraceptives.

SI conversion factors: to convert CRP to nmol/L, multiply by 9.524.

P values: t-test for continuous variables, chi-square test for categorical variables.

^a^ Arithmetic mean (SE).

^b^ For men 0.1 – 39.9 g/day and ≥ 40 g/day; for women 0.1 – 19.9 g/day and ≥ 20 g/day.

^c^ Only for women aged ≥ 50 years with no current use of OC (incident diabetes: 539; incident coronary events: 563).

^d^ Only for women aged < 50 years with no current HRT (incident diabetes: 358; incident coronary events: 362).

^e^ Geometric mean (antilog of SE).
