# Supplementary material for: Immunological and Cardiometabolic Risk Factors in the Prediction of Type 2 Diabetes and Coronary Events: MONICA/KORA Augsburg Case-Cohort Study
Source: PLoS One. 2011 Jun 6;6(6):e19852. doi: 10.1371/journal.pone.0019852 (PMC3108947; doi:10.1371/journal.pone.0019852)
Supplement: Table S3 — Correlation of inflammation-related biomarkers (Spearman correlation coefficients r) in the randomly sampled subcohort (n = 1,795). (DOC) [file pone.0019852.s005.doc]

**Table S3.** Correlation of inflammation-related biomarkers (Spearman correlation coefficients *r*) in the randomly sampled subcohort (n = 1,795).

|  | **CRP** | **IL-6** | **IL-18** | **TGF-β1** | **MIF** | **MCP-1** | **IL-8** | **IP-10** | **RANTES** | **Adiponectin** | **Leptin** | **sE-selectin** |
| --- | --- | --- | --- | --- | --- | --- | --- | --- | --- | --- | --- | --- |
| **CRP** | - | - | - | - | - | - | - | - | - | - | - | - |
| **IL-6** | 0.460 ^b^ | - | - | - | - | - | - | - | - | - | - | - |
| **IL-18** | 0.105 ^b^ | 0.090 ^a^ | - | - | - | - | - | - | - | - | - | - |
| **TGF-β1** | 0.019 | 0.019 | -0.024 | - | - | - | - | - | - | - | - | - |
| **MIF** | 0.092 ^b^ | 0.054 ^a^ | 0.020 | 0.029 | - | - | - | - | - | - | - | - |
| **MCP-1** | 0.081 ^a^ | 0.092 ^b^ | 0.265 ^b^ | 0.019 | -0.003 | - | - | - | - | - | - | - |
| **IL-8** | 0.135 ^b^ | 0.160 ^b^ | 0.226 ^b^ | 0.113 ^b^ | 0.041 | 0.301 ^b^ | - | - | - | - | - | - |
| **IP-10** | 0.210 ^b^ | 0.178 ^b^ | 0.238 ^b^ | -0.055 ^a^ | -0.014 | 0.184 ^b^ | 0.246 ^b^ | - | - | - | - | - |
| **RANTES** | 0.087 ^a^ | 0.086 ^a^ | 0.016 | 0.444 ^b^ | -0.111 ^b^ | 0.124 ^b^ | 0.138 ^b^ | 0.090 ^a^ | - | - | - | - |
| **Adiponectin** | -0.135 ^b^ | 0.117 ^b^ | -0.086 ^a^ | -0.074 ^a^ | -0.077 ^a^ | -0.011 | -0.035 | 0.003 | -0.027 | - | - | - |
| **Leptin** | 0.271 ^b^ | 0.127 ^b^ | 0.005 | -0.020 | 0.008 | -0.001 | -0.009 | 0.082 ^a^ | 0.041 | 0.194 ^b^ | - | - |
| **sE-selectin** | 0.180 ^b^ | 0.218 ^b^ | 0.104 ^b^ | 0.065 ^a^ | 0.050 ^a^ | 0.084 ^a^ | 0.110 ^b^ | 0.142 ^b^ | 0.021 | -0.162 ^b^ | 0.023 | - |
| **sICAM-1** | 0.247 ^b^ | 0.230 ^b^ | 0.067 ^a^ | 0.006 | 0.091 ^a^ | 0.034 | 0.129 ^b^ | 0.150 ^b^ | 0.043 | -0.074 ^a^ | -0.015 | 0.300 ^b^ |

^a^ p < 0.05; ^b^ p < 0.01.
