# Supplementary material for: Immunological and Cardiometabolic Risk Factors in the Prediction of Type 2 Diabetes and Coronary Events: MONICA/KORA Augsburg Case-Cohort Study
Source: PLoS One. 2011 Jun 6;6(6):e19852. doi: 10.1371/journal.pone.0019852 (PMC3108947; doi:10.1371/journal.pone.0019852)
Supplement: Table S4 — Predictive value of Cox regression models for each inflammation-related biomarker assessed by ΔAIC, IDI and NRI for incident type 2 diabetes. (DOC) [file pone.0019852.s006.doc]

**Table S4.** Predictive value of Cox regression models for each inflammation-related biomarker assessed by ΔAIC, IDI and NRI for incident type 2 diabetes.

| **Biomarker** | **ΔAIC1 ^a^** | **ΔAIC2 ^b^** | **IDI1**  **(95% CI) ^a^** | **IDI2**  **(95% CI) ^b^** | **NRI1 ^a^** | **NRI2 ^b^** |
| --- | --- | --- | --- | --- | --- | --- |
| None | --- | --- | --- | --- | --- | --- |
| **hsCRP** | 6.6 | -2.0 | 0.001  (-0.000-0.006) | 0.000  (-0.001-0.003) | 0.034 | -0.005 |
| **IL-6** | 0.0 | -1.2 | **0.001**  **(0.000-0.005)** | 0.001  (-0.000-0.007) | -0.002 | 0.000 |
| **IL-18** | 2.5 | 1.9 | **0.001**  **(0.000-0.022)** | **0.002**  **(0.000-0.032)** | 0.015 | 0.006 |
| **TGF-β1** | 3.5 | 2.0 | **0.001**  **(0.000-0.005)** | 0.001  (-0.000-0.008) | 0.020 | 0.031 |
| **MIF** | -0.1 | -1.8 | 0.001  (-0.000-0.003) | 0.001  (-0.000-0.003) | -0.006 | 0.008 |
| **MCP-1** | 4.7 | 1.0 | 0.001  (-0.000-0.005) | 0.001  (-0.000-0.008) | -0.006 | -0.018 |
| **IL-8** | -2.0 | -1.4 | 0.001  (-0.000-0.002) | 0.001  (-0.000-0.003) | 0.000 | 0.000 |
| **IP-10** | 1.8 | 2.8 | 0.006  (-0.000-0.032) | 0.013  (-0.000-0.034) | -0.017 | 0.001 |
| **RANTES** | 4.7 | -1.7 | **0.001**  **(0.000-0.005)** | 0.001  (-0.001-0.004) | 0.020 | 0.011 |
| **Adiponectin** | 171.3 | 78.0 | **0.036**  **(0.023-0.054)** | **0.021**  **(0.008-0.044)** | 0.312 | 0.126 |
| **Leptin** | 123.0 | 3.8 | **0.028**  **(0.017-0.052)** | 0.002  (-0.000-0.014) | 0.172 | 0.021 |
| **sE-selectin** | 98.1 | 59.8 | **0.027**  **(0.015-0.054)** | **0.025**  **(0.008-0.056)** | 0.174 | 0.073 |
| **sICAM-1** | 22.8 | 5.8 | **0.009**  **(0.002-0.020)** | 0.004  (-0.000-0.016) | 0.057 | 0.047 |
| **With all 13 biomarkers** | 340.7 | 139.8 | **0.098**  **(0.078-0.153)** | **0.061**  **(0.043-0.116)** | 0.586 | 0.202 |
| **With IL-18, adiponectin, sE-selectin, sICAM-1 ^c^** | 278.9 | 136.3 | **0.078**  **(0.058-0.117)** | **0.053**  **(0.030-0.097)** | 0.462 | 0.163 |

Bold print denotes statistical significance for IDI (*P*<0.05). “–0.000” denotes values between –0.0005 and 0.0000.

^a^ Adjusted for age, sex and survey (model 1).

^b^ Adjusted for age, sex, survey, BMI, systolic blood pressure, ratio of total cholesterol/HDL cholesterol, smoking, alcohol, physical activity and parental history of diabetes (model 2).

^c^ With biomarkers that were significantly associated with incident type 2 diabetes in multivariable-adjusted models (IL-18, adiponectin, sE-selectin, sICAM-1).

ΔAIC, IDI and NRI denote the differences between the model with the respective inflammation-related biomarker and the model without any inflammation-related biomarker. Values for differences between the model adjusted for age, sex, survey and cardiometabolic risk factors (model c) and the basic model adjusted for age, sex and survey (model a): ΔAIC=346.2, IDI=0.090, NRI= 0.537.
