# Supplementary material for: Immunological and Cardiometabolic Risk Factors in the Prediction of Type 2 Diabetes and Coronary Events: MONICA/KORA Augsburg Case-Cohort Study
Source: PLoS One. 2011 Jun 6;6(6):e19852. doi: 10.1371/journal.pone.0019852 (PMC3108947; doi:10.1371/journal.pone.0019852)
Supplement: Table S6 — NRI for inflammation-related biomarkers added to prediction models for incident type 2 diabetes and incident coronary events. (DOC) [file pone.0019852.s008.doc]

**Table S6.** NRI for inflammation-related biomarkers added to prediction models for incident type 2 diabetes and incident coronary events.

| Outcome | **Model** | **Biomarkers** | **Cut-off values for risk categories (%)** | | |
| --- | --- | --- | --- | --- | --- |
|  |  |  | 0 to <2, 2 to <5, 5 to <10, ≥10 | 0 to <3, 3 to <8, 8 to <15, ≥15 | 0 to <5, 5 to <10, 10 to <20, ≥20 |
| Incident type 2 diabetes | NRI1 ^a^ | With all 13 biomarkers | 0.598 | 0.586 | 0.515 |
|  |  | With IL-18, adiponectin, sE-selectin, sICAM-1 ^d^ | 0.493 | 0.462 | 0.472 |
|  | NRI2 ^b^ | With all 13 biomarkers | 0.198 | 0.202 | 0.230 |
|  |  | With IL-18, adiponectin, sE-selectin, sICAM-1 ^d^ | 0.182 | 0.163 | 0.220 |
| Incident coronary events | NRI1 ^a^ | With all 13 biomarkers | 0.097 | 0.050 | 0.049 |
|  |  | With IL-6, sICAM-1 ^e^ | 0.037 | 0.076 | 0.013 |
|  | NRI2 ^c^ | With all 13 biomarkers | 0.060 | 0.028 | 0.063 |
|  |  | With IL-6, sICAM-1 ^e^ | 0.038 | 0.025 | 0.052 |

^a^ Adjusted for age, sex and survey (model 1).

^b^ Adjusted for age, sex, survey, BMI, systolic blood pressure, ratio of total cholesterol/HDL cholesterol, smoking, alcohol, physical activity and parental history of diabetes (model 2).

^c^ Adjusted for age, sex, survey, BMI, systolic blood pressure, ratio of total cholesterol/HDL cholesterol, smoking, alcohol, physical activity, parental myocardial infarction and prevalent diabetes (model 2).

^d^ With biomarkers that were significantly associated with incident type 2 diabetes in multivariable-adjusted models (IL-18, adiponectin, sE-selectin, sICAM-1).

^e^ With biomarkers that were significantly associated with incident coronary events in multivariable-adjusted models (IL-6, sICAM-1).
